# Supplementary material for: Are carbon emissions trading and green financial instruments synergistic? -Comprehensive quantitative research based on content analysis
Source: PLoS One. 2024 Mar 7;19(3):e0298601. doi: 10.1371/journal.pone.0298601 (PMC10919601; doi:10.1371/journal.pone.0298601)
Supplement: S1 Data — (ZIP) [file pone.0298601.s001.zip › Data/地级市Z_F工作报告.zip!/─■╧─/╓╨╬└╩╨/└┤╫╘╣1⁄2╓┌║┼í╛┬φ┐╦╩2╛▌═°í┐_2018.docx]

各位代表：

现在，我代表市人民政府，向大会作工作报告，请予审议，并请政协委员和列席人员提出意见。

2017年工作回顾

天道酬勤，日新月异。2017年，党的十九大胜利召开，确立了习近平新时代中国特色社会主义思想的历史地位，描绘了决胜全面建成小康社会、夺取新时代中国特色社会主义伟大胜利的宏伟蓝图。自治区第十二次党代会作出了实施“三大战略”“五个扎实推进”的重大部署，明确了实现经济繁荣、民族团结、环境优美、人民富裕，与全国同步建成全面小康社会的奋斗目标。市委四届四次全会勾勒了“转型追赶、高质量发展”、坚决打好“三大攻坚战”、大力实施“三大战略”中卫方案、全面落实“五个扎实推进”重点任务、为决胜全面建成小康社会而不懈奋斗的美好愿景。

一年来，在自治区党委、政府和市委的坚强领导下，在市人大、政协的监督支持下，全市认真学习贯彻党的十九大精神，全面落实自治区第十二次党代会和市委各项决策部署，坚持稳中求进工作总基调，以深化供给侧结构性改革为主线，以“6+4”重点工作为抓手，攻坚克难，务实苦干，统筹推进稳增长、调结构、促改革、惠民生各项工作，较好完成了市四届人大一次会议确定的各项目标任务，迈出了“十三五”承上启下的关键一步。预计实现地区生产总值370亿元，增长7.5%左右；全社会固定资产投资362亿元，与上年持平；地方财政一般公共预算收入23.9亿元，同口径增长14.5%；社会消费品零售总额72.8亿元，增长10.6%；城镇、农村常住居民人均可支配收入分别达到25278元和9377元，增长8.6%和8.7%。

产业结构加快调整。现代农业优化升级。新建高效节水灌溉6.5万亩，粮食总产量6.7亿公斤。建设硒砂瓜品质品牌保护示范区40万亩，产值达15.2亿元。种植富硒苹果14.8万亩。建成万头奶牛养殖场3个、永久性蔬菜基地1万亩。“1+5”特色优势农业产值占比达87%。培育市级农业产业化龙头企业20家、农业合作社15家，农产品加工总产值增长5.2%。预计第一产业完成增加值52.4亿元，增长4%。新型工业加速推进。4家50MW光伏电站并网发电，宁钢60万吨高速线材、利安隆抗氧化剂、利丰年产300万件民族服饰等项目建成投产，中化锂电池新材料、江苏振发新能源组件等项目开工建设。培育认定“专精特新”中小企业26家，淘汰煤炭、钢铁、电石等落后产能85万吨，取缔“地条钢”生产企业4家。预计规模以上工业增加值增长8.2%。全域旅游加快发展。沙坡头南岸半岛生土度假村、沙坡头游客中心、沙坡头盛典演艺等项目进展顺利，完成旅游投资12.5亿元。建成“云天中卫”产业创新中心，开发中卫网景游戏“冬日威胁”，新建乡村旅游点7个。成功举办大漠黄河国际旅游节、中国全域旅游暨文旅特色小镇创新高峰论坛等重大节事活动。游客接待人次和旅游收入分别增长24%和26%。云天产业翘楚云集。美国亚马逊云计算中卫合作项目正式上线运营，奇虎360数据中心投入运营，中国移动数据中心即将投用，美利云数据中心二期、炫云数据中心等项目开工建设，国家旅游局、中国残疾人福利基金会、风云气象卫星等数据中心落户中卫。以PPP合作模式建设“12345”政务服务热线和“以克论净”环卫云、智慧医疗云、“多规合一”信息平台等云应用项目。启动军民融合创新示范区建设，西部军民两用飞艇基地、高分辨率对地观测系统宁夏应用中心等项目加快推进。“天地一体化无线电信号监测关键技术研究”项目列入自治区重大科技专项。云计算和军民融合产业及配套项目累计完成投资33.6亿元。交通物流破题发展。成功开行中卫—欧洲、中卫—俄罗斯国际班列，并实现常态化。沙坡头机场航线增至6条，实现旅客吞吐量15.9万人次，增长55%。中卫综合物流园区、中卫铁路口岸建设顺利推进，中宁国际陆港投入运营，天元锰业保税仓库通过验收，实现进出口额14.8亿元，增长27.7%。新建农村公路302公里。金融信贷稳健运行。人民币存贷款余额分别增长11.9%和17%，扶贫小额信贷不良率仅为0.05%。预计第三产业增加值增长8.5%。

生态环境稳步改善。出台“生态立市16条”，实施“蓝天碧水·绿色城乡”专项行动，开展大气污染“七项攻坚行动”，淘汰燃煤锅炉157台、黄标车1208辆，完成自治区下达任务的188.6%和115%，工业热气和城市供暖实现集中供应。全面建立四级“河长制”工作体系，综合整治黄河支流、入黄排水沟和城市黑臭水体，工业园区中水回用项目建成试运行，中卫市第一、第二污水处理厂提标升级改造等项目投入运营，黄河中卫段III类良好水质以上断面达100%。坚决整改中央第八环保督察组反馈问题，腾格里沙漠污染后续治理、沙坡头和南华山2个国家级自然保护区综合整治取得扎实成效。实施黄河卫宁过境段水生态治理与保护、水源涵养林、生态经济林、城市绿化等生态工程，完成人工造林26.4万亩，森林覆盖率达12.9%。深入实施城市美化亮化“九大工程”，新建、续建道路26条，铺设供热主管网27.2公里，累计建设公共租赁住房3.9万套，分配入住2.7万套，改造棚户区住房1.1万套、老旧小区31个。巩固提升城市深度保洁成果，“以克论净·深度清洁”项目被住房和城乡建设部评为中国人居环境范例奖，全区“城市双修”工作现场推进会在中卫召开。开工建设美丽小城镇6个，整治美丽村庄43个，改造农村危房1.03万户。沙坡头区迎水桥镇姚滩村入选全国改善人居环境示范村。

精准脱贫扎实推进。出台“脱贫富民28条”，大力实施“五项脱贫行动计划”，年内脱贫销号41个村，减贫3.93万人，贫困发生率从11.1%下降到7.9%。对建档立卡户实行动态管理，新识别贫困户1275户4760人。创新“华润基础母牛银行”“飞牛飞羊”金融扶贫模式，累计引进基础母牛1.7万头，赊销1.4万头，惠及群众5930户。设立2.4亿元扶贫产业担保基金，出台建档立卡贫困户“扶贫保”等政策，累计发放扶贫小额信贷11.9亿元，覆盖率达74%，户均贷款4.1万元。全市村级互助资金运行规模8000万元，1.05万农户受益。整合资金14.1亿元，69个贫困村实现“五通八有”目标。统筹推进移民搬迁工作，新建或回购移民住房2476套，搬迁安置移民8475人。加强与福建漳州对口扶贫协作，海原闽宁纺织工业园建成投用。

民生改善持续加强。坚持将70%以上的财力用于改善民生，12件民生实事全部办结。深化大众创业、万众创新，出台支持鼓励创新创业优惠政策，发放创业担保贷款1.5亿元，培育孵化基地7个，新增城镇就业8219人，转移农村劳动力15.5万人，城镇登记失业率控制在3.3%。沙坡头区、中宁县和海原县城乡居民基础养老金分别提高20元、35元和15元。优先发展教育，新改扩建幼儿园33所，学前三年教育毛入园率达82.6%。改造义务教育薄弱学校31所2.6万平方米，海原县义务教育均衡发展工作顺利通过国家评估认定。提升医疗卫生条件，建成市人民医院医技楼、市疾病预防控制中心大楼，迁建市中医医院、海原县中医医院等，建设60所标准化村卫生室。启动家庭医生签约服务和跨省异地就医结算。全面实施两孩政策，人口自然增长率9.29‰，出生政策符合率95.2%。沙坡头区成功创建为自治区级妇幼健康优质服务示范区。深入实施文化惠民工程和文化“六进”活动，在全区率先推行乡镇综合文化站“公建民营公助”管理运行模式，132个村级综合文化服务中心、市民健身步道等项目建成投用。成功举办十六届环青海湖国际公路自行车赛中卫赛段、首届全区大漠健身运动大赛等体育赛事。市文化体育新闻出版广电局被评为全国群众体育先进单位。深化“平安中卫”建设，深入推进“七五”普法，加强公共法律服务，加大矛盾纠纷排查化解力度，全市信访形势稳定向好。建成安全风险控制和隐患排查治理双重预防体系、安全生产在线教育服务平台，安全风险防范能力和从业人员整体素质明显提升。高质量通过国务院安委会4轮巡查检查，持续开展安全生产大检查，安全生产形势总体平稳。强化食品药品市场监管。深化民族团结进步创建，依法管理宗教事务，民族团结、宗教和顺、社会和谐的良好局面不断巩固。

改革开放不断深化。市委确定的76项改革任务顺利推进。深化“放管服”改革，全面实行告知承诺制，精简证照事权421项，推进实体政务大厅与网上办事大厅融合发展，60%的行政审批实现“不见面、马上办”“最多跑一次”目标。加快“三十三证合一”等商事制度改革，新登记企业3126户，增长12%。完成农村土地承包经营权确权登记颁证，县（区）均成立农村产权流转交易中心，农村产权抵押贷款规模达1.85亿元。深化投融资体制改革，吸引社会资本150亿元投入基础设施建设。加快工业园区电力体制改革，整合中卫工业园区电力设施，40家工业企业累计直接交易电量134.6亿千瓦时，降低成本3亿元。加快城市综合执法管理体制改革，将市城市建设监察支队整建制划转沙坡头区。稳步推进公立医院改革，全面取消公立医院药品加成，全年减轻患者医药负担3500万元。深入开展“项目建设年”“招商引资年”活动，推进全国工商联十一届十次常委会和中阿博览会签约项目落实，组织开展冬季招商引资大会战，全年招商引资到位资金304亿元。

政府效能明显提升。严格执行“三重一大”事项集体决策规定，认真执行人大及其常委会的决议、决定，主动接受人大工作监督、法律监督，自觉接受政协民主监督，办理人大代表建议19件、政协委员提案117件、办理答复率均为100%。修改、废止政府规范性文件186件。全面开展全国行政执法“三项制度”试点，在市县乡三级政府及行政执法部门普遍建立法律顾问制度，依法成立中卫仲裁委员会。扎实开展“两学一做”学习教育常态化制度化，严格落实全面从严治党各项制度，自觉执行党风廉政建设责任制。坚持“零容忍”惩治腐败，加强审计监督，开展项目资金管理专项整治，加大行政监察和整治“四风”力度，全市纪检监察机关受理信访举报484件，立案347件，给予党政纪处分344人。

一年来，我们扎实推进国防动员、双拥优抚等工作，发放退役士兵自主就业补助1312万元、现役军人家属优待金625.6万元，促进军民融合深度发展。外事、侨务、统计、新闻、出版、慈善、老龄、妇女儿童、青少年、残疾人、红十字会等事业取得新进步，地震、地质、人防、气象、档案、文史、社科等工作实现新发展。

“社会主义是干出来的”。在建设“中卫梦·凤凰城”的征程中，我们势破“胡焕庸线”，践行“总理三问”，2017年共同办成了几件事关中卫长远发展的大事、实事。在打造“云天中卫”方面，历时四年的美国亚马逊云计算合作数据中心正式上线运营，开创了“大云西移”“前店后厂”的国际先例，开创了世界500强产业扶贫新模式，在国内外将产生深远影响，中卫正成为全球新一代云计算产业集聚区。美利纸业公司实现华丽转身，市值居宁夏之首，成为央企混合所有制改革的典范。在全域旅游方面，大力创建全国首批全域旅游示范城市，中卫市被评为国家级旅游业改革创新先行区和全国“厕所革命”十大优秀城市之一。在交通物流方面，中卫至吴忠城际铁路、中卫至兰州客运专线加速推进，中卫人民期盼已久的“高铁梦”正在变为现实。在特色产业方面，“中宁枸杞”异军突起，成功入选中国农产品地理标志，品牌价值跃升为161.6亿元，“中宁枸杞走进千家万户”正在变为现实。“香山硒砂瓜”荣获全国“一村一品”十大知名品牌。天元锰业与亨通集团实现战略合作，成立300亿元产业基金。行业龙头明盛染化浴火重生，占领世界65%市场份额。在脱贫攻坚方面，市财政筹措1145万元，对2290名在校贫困大学生每人补助5000元，让每一个贫困家庭学生圆了大学梦。宁夏中部干旱带西部供水工程提前通水，中宁县喊叫水扬水工程顺利推进，农村自来水覆盖率达95%、自来水入户率达83.2%，结束了当地群众长年喝“苦咸水”“雨窖水”的历史。

事非经过不知难。在肯定成绩的同时，我们也清醒地看到，我市经济社会发展中仍面临不少困难和问题。主要表现在：产业总量不足且结构调整任务繁重，新旧动能正在转换，内生增长机制还未形成；山川、城乡、区域发展不均衡、不协调的问题较为突出，深度脱贫攻坚任务艰巨；财政收入总量小与公共服务、民生保障等刚性支出增加的矛盾依然突出；各类人才匮乏，特别是“高精尖”人才严重不足等等。这些问题，我们必须高度重视，采取有力措施，切实加以解决。

2018年主要目标和重点任务

今年是贯彻党的十九大精神的开局之年，是改革开放40周年，是决胜全面建成小康社会的关键一年，也是自治区成立60周年。做好今年工作，意义重大。

政府工作的总体要求是：高举中国特色社会主义伟大旗帜，以习近平新时代中国特色社会主义思想为指导，全面贯彻党的十九大、中央经济工作会议精神，按照自治区第十二次党代会、自治区党委十二届二次、三次全会及市第四次党代会的部署，围绕“中卫梦·凤凰城”，坚持稳中求进工作总基调，紧紧抓住“转型追赶、高质量发展”这条主线，不忘初心、牢记使命，振奋精神、实干兴卫，坚决打好“三大攻坚战”，大力实施“三大战略”中卫方案，全面落实“五个扎实推进”重点任务，实施工业转型升级、特色农业提质增效、培育经济发展新动能、城乡山川协调发展“四项行动计划”，为决胜全面建成小康社会、夺取新时代中国特色社会主义伟大胜利而不懈奋斗。

主要预期目标是：力争全市地区生产总值增长8%，全社会固定资产投资增长8%左右，地方财政一般公共预算收入增长8%，社会消费品零售总额增长9%，城镇、农村居民人均可支配收入分别增长8%和8.5%，城镇登记失业率控制在4%以内，科技研发经费占GDP比重提高到1.02%左右，节能减排和环境质量改善完成国家和自治区下达任务。

实现上述目标，我们将抓好五项重点工作：

一、坚持创新驱动，加快产业转型

深化供给侧结构性改革，加快新旧动能转换，不断提高供给质量，推动中卫经济高质量发展。

做优做特现代农业。聚焦“1+5”特色优势产业，支持发展“一县一品”，发挥“枸杞之乡”“硒砂瓜之乡”“马铃薯之乡”优势，带动农民持续稳定增收。强化原产地保护意识，推进枸杞产业标准化、规模化、集约化发展。全市新种植枸杞3.4万亩。制定枸杞产业生产技术、产品质量标准，加大品质品牌创建保护力度，加快“三品一标”认证，打造中宁枸杞自主品牌，力争枸杞产业综合产值突破100亿元。加快富硒产业3.0研究中心和富硒产业试验基地、种植基地、加工基地“一中心三基地”建设，建成硒砂瓜万亩小产区6个，种植苹果0.4万亩，推进江苏绿港现代农业示范基地、九晟“五优”设施蔬菜标准化生产园区建设。成立中卫富硒农产品股份公司，建设富硒产业市场运营平台，打造“中国塞上硒谷”。加快制定畜牧产业发展规划，提升沐沙、阜民丰等万头奶牛养殖场存栏规模和科技装备水平，把沙坡头区打造成全区奶产业大县。以现有龙头企业为主，引导扶持企业加快发展，全市奶牛存栏达6.5万头，肉牛、肉羊饲养量分别达到35万头和260万只。充分发挥华润、万齐、香岩、玺赞等重点龙头企业作用，带动优势特色产业做大做强，提升产业整体效益，集中力量新培育龙头企业10家以上。

培育壮大战略产业。加快发展云计算产业，依托云计算优势，拓展人工智能、区块链、虚拟现实、物联网等新兴产业。发挥亚马逊“头雁效应”，加快推进美国亚马逊云计算中卫合作项目扩容，启动二期建设，力促美利云二期、金融云等数据中心建成投运，开工建设中国联通、炫云、天地通等数据中心，实施智慧消防云基地建设项目，争取国家部委、央企数据灾备中心落户中卫，力争服务器规模达到20万台。引进美国ZT、德国世图兹等一批云存储、云制造项目，打造云计算上下游产业链。加快政务云、医疗云、环保云、旅游云等云应用项目建设，打造一体化政务服务信息平台。加强与京东、阿里等大型互联网企业的沟通洽谈，力促更多企业云服务业务在中卫集结。加快发展军民融合产业，争取设立军民融合产业发展基金，争创国家级军民融合创新示范区。围绕“一中心、四基地、一展会”的目标，加快建设一体化军民融合大数据中心，争取卫星遥感和气象通信、导航等一批数据中心落户中卫。加快打造航天产业基地，规划建设射电天文观测站、卫星数据接收天线组阵、卫星定标和真实性检验场，力争发射“宁夏一号”卫星。加快打造航空产业基地，在建成飞艇基地的基础上，开拓飞艇应用市场，争取低空开放，积极发展通航产业。加快打造成果转化基地，实现火冰灭火剂等军民两用产品在中卫研发、组装和生产，扩大规模、占领市场。以推进武器装备试验分靶场、星空体验等项目为契机，建设新型国防教育基地。积极规划筹办中国防务装备展。加快发展新材料产业，鼓励今飞轮毂、锦宁巨科等现有铝基产业开发新产品、增加新产能。积极引进锰基复合材料和亨通三元正极材料落地，培育形成从锰材料、复合材料到制造的产业链条。编制能源产业发展规划，重点以振发光伏模板装备制造、银阳、协鑫单晶拉棒、电池组件等项目为龙头，加快建设风力、光伏发电项目，打造加工、制造、储能、应用全产业链的新能源产业基地，鼓励新能源企业走出国门开拓海外市场。

改造提升传统产业。以优化存量、培育增量为重点，加快传统产业改造升级，规上工业增加值增长8.5%。围绕煤炭、电力、冶金、化工、建材等传统产业，补链、延链、强链，推进产业向高端化、集群化方向发展。加快中化锂电池材料、三元中泰低铝合金炉等项目达产达效，力促中铁凤凰城智能制造、宁夏润华20万吨储能熔盐、宁夏紫光蛋氨酸三期等重点项目开工建设。积极引导钢铁、金属锰、电解铝、铁合金等传统产业兼并重组、改造提升，推进节约技改和节能新技术新产品推广应用，淘汰化解落后和过剩产能。加快推动中卫中宁工业园区整合发展，组建卫宁经济技术开发区，启动编制规划，推进扩区调位，优化要素配置，加快园区循环化改造。统筹做好产业布局和基础设施配套，着力打造光伏新能源、云计算及军民融合、精细化工3个百亿元级和新材料1个千亿元级的产业集群。

提档发展现代服务业。进一步丰富完善“一核、两翼、三带、四区、六线、多节点”旅游规划布局，拓宽旅游发展新空间，加快全域旅游示范市建设。突出抓好沙漠沙世界、太阳酒店、星星酒店为重点的沙坡头新北区建设，加快黄河生态旅游带、高庙历史文化街区二期等项目建设，实施沙坡头新北区骨干道路、景区间云轨交通项目，全市旅游业投资力争达到36亿元以上。高庙景区免费向游客和市民开放。推动“旅游+”融合发展，打造云产业体验、工业旅游、富硒文化、生态农庄、康养旅游、以《沙坡头盛典》为代表的旅游演艺等，持续放大中卫旅游差异化效应。建成卧龙酒店等一批星级酒店，提升服务质量和标准。深化与海南三亚、福建漳州等地的区域合作，举办大漠黄河国际旅游节等节会赛事活动，加大旅游包机、旅游专列扶持力度，吸引更多旅客集聚中卫。全年接待游客突破800万人次，旅游总收入达到75亿元，分别增长20%以上。加快商品房去库存，推行棚改货币化安置政策，规范住房市场开发、销售、中介等行为，保持房地产平稳健康发展。在北京、台北等地建设中卫特色产品外埠展销窗口，加快建设农村电商、社区便民商业、冷链物流体系，完善各类商业网点布局，打造特色商业街区，提升商贸流通水平。设立服务业发展引导基金，推动服务业发展提速、比重提高、水平提升。

提升科技支撑能力。抢抓沿黄科技创新改革试验区建设机遇，设立创新创业引导基金，加大科研投入，确保2018年起科技研发经费投入年均增长30%以上。大力培育创新型企业、科技小巨人企业和国家高新技术企业，贯通科技型企业成长通道。充分发挥企业创新主体作用，引导企业进行原始创新、集成创新、引进消化吸收再创新，加快科技成果转化应用，支持紫光蛋氨酸碱纳法工艺、西云数据新一代全自然风冷、协鑫集团高效单晶等一批国际领先水平的技术产业化应用。成立中卫创新发展研究院，积极与国内外科研院所、知名高校开展科研、人才等交流合作，搭建“政产学研用”五位一体协作创新平台，支持宁夏大学筹办亚马逊云计算学院、国际旅游学院、交通物流学院，创新校地、校企合作新机制。推进东西部科技合作，实施好“天地一体化无线电信号监测关键技术研究”“载重、平流层飞艇”等项目。出台引进高校毕业生特别是重点院校硕士、博士的激励政策，吸引一批符合中卫发展需要的高层次人才和创新创业团队。促进科技与金融结合，扩大科技型企业风险补偿资金贷款规模。全市高新技术企业累计达5家、自治区级科技型中小企业达60家，知识产权示范、优势、试点企业达15家，各类科技创新平台20个以上。

二、坚持脱贫富民，增强人民福祉

始终把人民利益摆在至高无上的位置，实施脱贫富民战略，办好10件民生实事，想群众之所想，急群众之所急，让人民生活更加幸福美满。

坚决打赢精准脱贫攻坚战。按照“六个精准”“五个一批”要求，实施三年滚动计划，既不降低标准、拖后延迟，也不吊高胃口、提标冒进，力争年内43个贫困村、2.82万人脱贫。集中优势力量攻坚深度贫困地区，财政专项存量资金、新增资金重点用于73个深度贫困村，统筹整合财政涉农资金和教育、卫生、民政等社会事业资金向深度贫困村倾斜，每个村整合资金不少于1000万元。实现建档立卡贫困家庭子女学前到高中教育免学费、常住人口1500人以上深度贫困村学前教育全覆盖。实施保险脱贫计划，为4.2万贫困户购买家庭意外伤害保险、为16.5万贫困人口购买大病补充医疗保险、为贫困户购买扶贫产业保险。深入推进“华润基础母牛银行”“飞牛飞羊”金融扶贫模式，充分发挥扶贫产业担保基金的撬动作用，力争发放扶贫小额贷款达11亿元，年末贷款余额增长10%，建档立卡贫困户贷款覆盖率达75%以上，户均贷款5万元。开工建设西海固地区脱贫引水工程，建成中南部城乡饮水安全海原片区连通工程。加快沙坡头区至海原、海原至平川等高速公路项目，推进海同高速尽快通车。持续抓好易地扶贫搬迁工作，市内安置移民682户3007人，市外安置418户1819人，全面完成“十三五”易地搬迁工作任务，加强后续产业扶持，力争搬迁一户、脱贫一户。深化闽宁对口扶贫协作，做细做实结对帮扶项目，形成脱贫攻坚社会合力。

织牢社会保障网。突出抓好高校毕业生、就业困难人员等重点人群就业和军队退役官兵的就业安置，购买公益性岗位200个，城镇新增就业8200人，农村劳动力转移就业14.5万人，高校毕业生总体就业率达85%以上。着力增加职工收入，按照中央、自治区政策要求，调整机关事业单位基本工资标准，落实地区附加津贴制度，完善公务员奖金制度，推进企业职工收入稳定增长，适当提高社会最低工资标准。落实好强农惠农政策，挖掘农业生产经营性收入，增加务工工资性收入，释放财产性收入红利，不断拓宽农民增收渠道。积极推进医养结合试点，加快建设中卫市慈爱康复中心，建成中卫市救灾物资仓库。实施全民参保计划，稳步提高各项社会保险参保覆盖面，推进“五险合一”服务平台建设。建成中卫市残疾人康复中心，全面落实困难残疾人生活补贴和重度残疾人护理补贴制度。推进公租房租售并举，完成公租房分配入住1.5万套，分配率达100%，让更多住房困难户住上“暖心房”。

提升公共服务水平。优先发展教育事业。加快发展普惠性学前教育，新改建农村幼儿园15个，切实解决学前幼儿“入园难”问题。优化教育资源配置，全面实施义务教育薄弱学校改造工程，加快教育信息化建设，加强教师队伍建设，健全完善覆盖学生资助体系，巩固义务教育均衡发展成果，推进城乡义务教育一体化发展。统筹普通高中和职业教育协调发展，高中阶段教育质量和普及水平持续提升。实施农村中小学取暖保障工程。推进健康中卫建设。完成市中医医院迁建、市妇幼保健计划生育服务中心业务用房扩建项目，建成市级危重孕产妇和新生儿救治中心。深化公立医院改革，加快分级诊疗进程。扎实开展爱国卫生运动，争创国家卫生城市。大力发展公共文化事业。深入实施文化惠民工程，推广“公建民营公助”模式，建好用好村级综合文化服务中心、农村文化大院，盘活乡镇文化资源，推动公共文化设施向基层延伸、向贫困地区倾斜。广泛开展群众性文体活动，推动全民健身与全民健康深度融合。鼓励支持精品文艺创作，宣传推介60大庆献礼剧《沙漠绿洲》。积极承办大型体育赛事。加快大麦地、中宁枸杞、海原民俗文化园区建设，不断发展“文化+”创意创新业态。加强文化遗产保护传承。全力做好第四次全国经济普查工作。创新社会治理。完善立体化社会治安防控体系，依法严厉打击和惩治黄赌毒黑拐骗等违法犯罪活动，保护人民人身权、财产权、人格权。牢固树立安全发展理念，健全公共安全体系，进一步完善安全生产责任制，加快推进安全生产领域改革和发展，坚决遏制重特大事故发生。健全应急救援体系，提升突发事件应急响应、处置和保障能力。加强国防动员和后备力量建设，提高应急应战能力。完善人防、交战、经动等配套设施和人员队伍建设。全面贯彻党的民族政策和党的宗教工作基本方针，深化民族团结进步教育，加强各民族交往交流交融，共同团结奋斗，共同繁荣发展。坚持我国宗教中国化方向，积极引导宗教与社会主义社会相适应。

三、坚持生态立市，建设美丽中卫

牢固树立绿色发展理念，严守生态保护、耕地保护红线和城镇建设用地扩展边界线，让中卫蓝天常在、碧水长流、空气常新。

加强生态建设。持续推进大六盘生态林、水源涵养林、生态经济林、北部防护林和农田林网建设，实施高速公路、城市园林、工业园区廊道和农村国道省道绿化等工程，落实禁牧封育、退耕还林各项政策，加快移民迁出区生态修复力度，防止草原风化沙化。全年完成营造林34.3万亩，森林覆盖率达13.9%，努力建设宁夏西部重要生态安全屏障。划定生态保护红线、永久性基本农田、城镇开发边界三条控制线，加强沙坡头、南华山两个国家级自然保护区的管护。落实生态补偿机制，加强林地资源管理和征地占用、木材加工流通等监管，严厉查处涉林违法行为。

加强环境保护。深入开展大气、水、土壤等污染防治，严格落实国家新一轮蓝天保卫战三年作战计划，扎实开展大气污染防治攻坚战，紧盯PM2.5、PM10，综合治理煤尘、扬尘、汽尘、烟尘，加快集中供热管网改造，全面取缔城区燃煤锅炉，加快工业集聚区工业燃煤锅炉改造，有序推进乡村集中供热。抓好建筑工地、城市道路、渣土运输、采矿区域的扬尘治理，巩固城市道路机械化清扫、喷雾降尘等综合降尘措施，淘汰黄标车老旧车，对重点区域或住户实施“煤改电”“煤改气”，空气优良天数比例达80%以上。深入实施“水十条”，全面落实河长制，坚持一河（湖）一策，加强黄河过境段、入黄排水沟、湖泊湿地、城市黑臭水体治理，实施沙坡头区第三排水沟综合整治工程，加快各县（区）污水处理厂建设，全面取缔企业直接入河（湖）排污口。确保重点河（沟）水质稳定达到IV类以上，黄河过境段水质稳定达到III类以上。全面实施“土十条”，有效治理农业面源污染，进一步提升工业固废的减量化、资源化、无害化处理水平，确保土壤安全。统筹抓好中央第八环保督察组反馈问题的整改，依法依规完成“绿盾”自然保护区整改任务。用高新技术改造传统产业，提高环保准入门槛，严格控制“两高一资”行业发展，不符合环评标准的一律不得落地。严格落实能源和水资源消耗、建设用地等总量和强度双控行动，促进各类资源节约高效利用。

加快市县融合。认真落实沿黄生态经济带各项部署，加快市县融合发展，推进沙坡头区和中宁县相向发展。按照“产业连城、生态连城、水系连城、交通连城”的思路，实施黄河中卫城市过境段水生态治理保护、中央大道水系及滨河北路水系湖泊连通整治等工程，提升区域整体竞争力。积极探索盘活海兴开发区闲置资产，加快形成新能源装备、特色农副产品加工和轻纺服装产业集群，打造新的经济增长点。大力实施乡村振兴战略，充分发挥城镇辐射带动作用，推进中宁县老城改造和海原县大县城建设，着力打造旅游新镇、华润希望小镇、富硒文旅小镇等一批特色小镇，引导人口、产业、资本向城镇聚集。

统筹城乡发展。加快新老城区、地上地下建设，新建续建市政道路22条23公里，改造棚户区住房6080套。加快沙坡头区高铁商圈基础设施建设，高标准改造中卫火车站，建设中宁天元热电厂集中供热管网及中水管网、海原运动广场等工程。实施人车路综合治理，加快城市路网、停车设施、充电站（桩）建设，推广新能源汽车运用，倡导绿色出行。加强农村环境综合整治，建设美丽小城镇3个、美丽村庄20个，实施危房改造4000户、农户改厕2000户。开展农村公路两旁、房前屋后增绿行动，建设美丽乡村。

四、坚持改革开放，激发内生动力

坚持以改革的思维、开放的理念谋划发展、推动发展，逢山开路，遇水架桥，着力破解制约发展的深层次问题，着力拓宽更加广阔的发展空间。

全面深化改革。深入推进“放管服”改革。严格落实“两个清单”制度，全面推广“互联网+政务服务”，年内80%行政审批服务事项实现“不见面，马上办”，建成“不见面”审批服务新模式，行政审批事项全部进入大厅，做到网上办、集中批、联合审、区域评、代办制、不见面，逐步实现“让群众最多跑一次”、进入一个窗口办妥一揽子事的目标。深化商事制度改革，扎实推进“多证合一”，降低市场准入门槛和成本，进一步激发市场活力。深化国资国企改革。推进国有企业归并重组改革，规范国资监管、国有企业融资运营平台，构建“两级授权、三层架构”的国资监管体系。完善企业法人治理结构，建立健全现代企业管理制度。深入实施国有企业负责人薪酬制度改革，稳妥推进国有企业混合所有制改革。创新投融资体制机制，探索政府与社会资本合作新模式，引导金融企业在卫增设分支机构，着力增强经济发展活力。进一步加强政府性债务管理，规范举债融资机制，积极消化存量债务，完善风险防控体系，严禁盲目举债、过度举债、违规举债，坚决遏制债务增量。深化农村改革。扩大农村产权登记颁证范围，推进农村“两权”抵押贷款试点，全市发放抵押贷款2亿元。抓好沙坡头区农村集体产权制度改革试点工作，成立土地股份制合作社10个。稳妥推进自然资源所有权确权登记，扩大湿地产权确权试点范围。推进工业园区电力体制改革，发挥售电公司市场能动作用，推行大用户电力直接交易，着力打造低成本工业园区。

持续扩大开放。主动融入“一带一路”建设，充分利用中阿博览会、内陆开放型经济试验区等重要平台，着力打造丝绸之路经济带战略支点。突出抓好吴忠至中卫城际铁路、中卫至兰州客运专线、乌玛高速等项目建设。完善现有物流园区基础设施配套，成立卫宁物流投资公司，加快卫宁物流园区融合发展，引进8—10家3A、4A级物流企业。重点推进海兴开发区核辐照站项目建设，构建农业龙头企业+物流+马铃薯等农产品集散地的发展格局，推动海兴物流产业发展。按照“一港四区”发展思路，推进中宁陆路口岸、中卫铁路口岸、中卫铁路综合货场、沙坡头机场航空口岸建设，开展铁路班列及多式联运合作。扩建沙坡头机场，继续增加航线航班，与更多省会城市实现直飞，旅客吞吐量突破20万人次。规划建设“苏商工业园”，以入园的苏化集团、扬农集团、中化国际等企业为载体，实现资源利益共享。启动网上丝路枢纽工程，积极拓展与信息丝路沿线国家的电子商务、技术合作、信息共享和文化交流服务。深化与周边地区合作，促进区域设施联通、产业互补。坚定不移地鼓励、支持、引导非公有制经济发展，优化融资环境，扶持民营企业做大做强做优，弘扬优秀企业家精神，构建“亲”“清”新型政商关系。坚持引资、引技、引智并重，加大精准招商力度，全面落实已签订的合作协议，提高项目履约率、资金到位率和投产达效率，招商引资到位资金300亿元。

五、坚持依法行政，建设高效政府

加快转变政府职能，创新管理服务方式，不断提升政府执行力、落实力和公信力。

提高政治站位。深入学习贯彻党的十九大精神，牢固树立“四个意识”，坚定“四个自信”，坚决维护党中央权威，坚决服从党中央集中统一领导，始终在思想上政治上行动上同以习近平同志为核心的党中央保持高度一致。严守政治纪律，坚定政治信仰，增强政治担当，全面提升执行力，努力增强政府公职人员的改革创新本领、科学发展本领、依法行政本领、群众工作本领、狠抓落实本领，更好担负党和人民赋予的重要职责。

始终依法行政。坚决服从市委领导，自觉接受市人大及其常委会的法律监督和工作监督、市政协的民主监督、社会舆论和人民群众的监督，认真办理人大代表建议、政协委员提案，及时回应人民群众关切。坚持“三重一大”事项向市委报告制度，坚持重大决策公开听证、专家论证、合法性审查、集体研究决定。继续推进行政执法“三项制度”，所有行政行为都要于法有据、程序正当、公正公平。严格制定规范性文件。做好行政复议和行政应诉工作，完善政府法律顾问制度，依法化解行政争议。扎实推进政务公开，健全政府新闻发布制度，保障群众的知情权、参与权和监督权。

始终求真务实。扎实开展“不忘初心、牢记使命”主题教育，打好作风建设持久战。大力弘扬立说立行、马上就办的作风，凡是自治区党委、政府和市委的决策部署，必须第一时间传达落实。注重调查研究，继续开展厅处级领导干部驻村蹲点调研，深入基层一线，问需于民，问计于民，为群众办实事、解难事。健全督查问责机制，加强政务督查和效能监督，完善常态化督查工作机制，坚决整治庸懒散慢拖和不作为、乱作为等问题，做到有责必问、有错必究。健全正向奖励机制，落实容错纠错制度，加大督查问责力度，营造肯干事、能干事、干成事的浓厚氛围。坚持说实话、谋实事、出实招、求实效，以钉钉子的精神抓落实，确保各项决策部署落地生根、开花结果。

始终勤政廉政。严格贯彻全面从严治党要求，认真落实《中国共产党廉洁自律准则》和《中国共产党纪律处分条例》，切实加强政府系统思想政治建设。严格落实“一岗双责”，加强对重大工程、重点项目、资金拨付、招投标等重点领域和关键环节的监督检查，扎紧制度笼子，确保资金安全、项目安全、干部安全。严格落实自治区《关于深入贯彻中央八项规定精神进一步加强和改进自治区党委常委会作风建设的若干意见》和市委相关规定，继续整治“四风”问题，坚决反对特权思想和特权现象。厉行勤俭节约，反对铺张浪费，政府带头过紧日子，带头压缩一般性支出。坚决纠正侵害群众利益的不正之风，坚持无禁区、全覆盖、零容忍惩治腐败，实现干部清正、政府清廉。

各位代表，潮平两岸阔，风正一帆悬，“不到长城非好汉”。让我们紧密团结在以习近平同志为核心的党中央周围，坚决贯彻落实自治区党委、政府决策部署，在市委的坚强领导下，进一步振奋精神，实干兴卫，为中卫转型追赶、高质量发展，实现经济繁荣、民族团结、环境优美、人民富裕，与全国全区同步建成全面小康社会不懈奋斗。
